# Above the line: oxygen overshoot in the oximetry trace

**DOI:** 10.1007/s44470-026-00183-8

**Published:** 2026-09-18

**Authors:** Ari R. G. Manuel

**Affiliations:** Department of Respiratory Medicine, Wye Valley NHS Trust, Hereford, UK

Cheyne-Stokes respiration produces a familiar crescendo-decrescendo pattern: oxygen saturation falls, recovers, and may rise above the preceding stable-sleep baseline. Sleep medicine has concentrated on the downward component. Hypoxic burden, which integrates the depth and duration of event-related desaturation, predicts cardiovascular outcomes better than a simple event count [1]. In their study recently published in the *Journal of Clinical Sleep Medicine*, Azarbarzin and colleagues turn attention above the line by quantifying oxygen overshoot burden: the cumulative area of the SpO_2_ trace above an individual’s stable-sleep baseline, normalized for sleep time [2].

In 7530 participants from the Sleep Heart Health Study and Osteoporotic Fractures in Men Study, 2258 major adverse cardiovascular events (MACE) occurred during nearly nine years of follow-up. Among participants with central sleep apnea and high overshoot burden, MACE occurred in 54.6%, compared with 25.6% of controls (adjusted hazard ratio, 1.45). Central sleep apnea with low overshoot burden did not differ from controls, and no corresponding association was seen in obstructive sleep apnea. The association persisted after adjustment for apnoea-hypopnoea index, central apnea index, and hypoxic burden and for estimated loop gain in MrOS [2]. The specificity is intriguing, but it does not distinguish marker from mediator.

The proposed oxidative-stress explanation needs greater caution. Oxygen ‘overshoot’ here is a relative oximetry signal, not demonstrated hyperoxia: an SpO_2_ rise above a person’s stable-sleep baseline does not establish an elevated PaO_2_or tissue oxygen exposure. The study provides no mechanistic pathway, oxidative biomarker, or bench or animal evidence showing that this specific signal causes oxidative injury. Evidence concerning intermittent hypoxia and reoxygenation cannot simply be transferred to this above-baseline signal [2]. Indeed, previous work in obstructive sleep apnea associated greater post-event SpO_2_overshoot with lower nocturnal glucose, not evidence of harm [3]. It is therefore premature to treat the oxygen rise itself as the causal exposure.

A more plausible interpretation is that the oximetric overshoot is the visible tail of a larger ventilatory-autonomic response. High post-apnea ventilatory drive causes hypocapnia and reflects unstable ventilatory control. Human apnea studies show marked sympathetic and blood pressure surges around event termination, followed by lung inflation-related vagal modulation during recovery [4, 5]. These autonomic oscillations offer a plausible link with MACE without requiring a modest saturation rise to be toxic.

This interpretation exposes an important analytical gap. Adjustment for event frequency and hypoxic burden did not establish that overshoot was independent of the severity of the respiratory event itself. A longer or more severe event can accumulate hypercapnia, chemoreflex drive, arousal-related and sympathetic activation, and loss of lung inflation-related vagal restraint; these stimuli may then generate a larger recovery breath and oxygen overshoot [5]. Desaturation does not fully represent them. In obstructive sleep apnea, ventilatory burden predicts cardiovascular outcomes and explains much of the variation in hypoxic burden [6]. Oxygen overshoot may likewise summarize otherwise hidden physiological load in central sleep apnea.

Residual cardiac confounding remains another possibility. In older community cohorts, central sleep apnea and Cheyne-Stokes respiration may reflect cardiac dysfunction, elevated filling pressure and prolonged circulation time rather than mediate their consequences [7]. Heart failure identified from self-report and clinical records may miss subclinical disease. Adjustment for loop gain in one cohort helps, but detailed cardiac phenotyping is still needed.

Two further cautions temper clinical translation. The central sleep apnea subgroup comprised only 303 participants, so confidence intervals matter more than the headline event proportions. The subject-specific baseline also outperformed a fixed threshold of SpO_2_at or above 96%, confirming that the result depends on small relative differences [1]. With cohorts that were 88% White, known differential pulse-oximeter error by skin pigmentation is relevant, although its effect on an above-baseline area is unknown [8].

The next study should measure event duration, airflow and effort, carbon dioxide, arousals and event-level autonomic responses alongside oximetry, with detailed cardiac phenotyping and representative recruitment. It should test whether oxygen overshoot adds prognostic information after those features are included. Until then, oxygen overshoot burden is a promising risk marker, not a demonstrated oxidative mechanism or treatment target.

## Data Availability

Not applicable.
